# Neither Parents’ Sex Nor the Type of Family Modulates Attentional Bias Toward Infant Faces: A Preliminary Study in Different-Sex and Same-Sex Parents

**DOI:** 10.1007/s10508-024-02875-9

**Published:** 2024-05-29

**Authors:** Micol Gemignani, Michele Giannotti, Paola Rigo, Paola Venuti, Simona de Falco

**Affiliations:** 1 Department of Psychology and Cognitive Science, University of Trento, Corso Bettini 84, 38068 Rovereto, TN Italy; 2 Department of Developmental Psychology and Socialisation, University of Padua, Padua, PD Italy

**Keywords:** Attention, Parenting, Same-sex parent family, Face processing, Infants, Sexual orientation

## Abstract

An attentional bias toward infant versus adult faces has been detected in parents and positively associated with sensitive caregiving behaviors. In previous research, the attentional bias has been measured as the difference in attention, in terms of reaction times, captured by infant versus adult faces; the larger the difference, the greater the cognitive engagement that adults deployed to infant faces. However, research so far has been mostly confined to samples of mothers, who have been more represented than fathers. Moreover, new family forms, especially same-sex families of men, have been left out of research. To clarify potential sex differences and extend previous findings to diverse family forms, we implemented a modified Go/no-Go attentional task measuring attentional bias to infant faces in parents with children aged from 2 to 36 months. The sample (*N* = 86) was matched and included 22 fathers and 22 mothers from different-sex families and 20 fathers and 22 mothers from same-sex families. Overall, the results confirmed that infant faces induced a greater attentional bias compared to adult faces. Moreover, we found that neither the type of family nor parents’ sex modulated the attentional bias toward infant faces. The findings are discussed in relation to the importance of understanding the correlates of parental response to infant cues going beyond a heteronormative perspective on parenting.

**Supplementary Information:**

The online version contains supplementary material available at 10.1007/s10508-024-02875-9.

## Introduction

The processing of infant faces inherently differs from that of adult faces (Caria et al., 2012). Infant face morphology is characterized by typical features, including large, bulbous forehead, large eyes, small chin, and narrow nose. Those characteristics form the so-called “baby schema” (Lorenz, 1943), which also includes infants’ sounds and smell. The caretaking mechanism triggered by baby schema and other infants’ features was referred to as “Kindchenschema” by Lorenz (1943), who defined it as an innate releasing mechanism for eliciting caregiving behaviors in adults.

The attentional bias to infant faces is one of the mechanisms described in adults in response to baby schema (Jia et al., 2022). In other words, it has been consistently found that adults’ attention prioritizes infant facial features over other stimuli (Brosch et al., 2007; Dudek & Haley, 2020; Gemignani et al., 2022, 2024; Oliveira et al., 2017; Pearson et al., 2010, 2011a, 2011b, 2013; Thompson-Booth et al., 2014a, 2014b). The attentional bias to infant faces has been generally measured, using cognitive conflict tasks (e.g., Stroop, Go/No Go, visual search tasks), as the difference in attention in terms of reaction times (RTs) captured by infant versus adult faces; the larger the difference, the greater the cognitive engagement that adults deployed to infant faces (Lucion et al., 2017). Remarkably, this cognitive mechanism has been identified as an early determinant of sensitive parenting and a factor associated with the quality of parent–child interactions (Dudek & Haley, 2020; Pearson et al., 2011a).

Given that the facial expression of infants conveys relevant information regarding their physical and mental states (Sullivan, 2014), past research on attentional bias to infant faces has focused on the modulating role of infants’ facial expressions (Lucion et al., 2017). While a significant effect of the type of emotional valence of faces was detected in some studies (i.e., a greater attentional bias was detected in response to infant distressed faces; Pearson et al., 2010, 2011a, 2011b, 2013), others did not find such an effect (Dudek & Haley, 2020; Gemignani et al., 2022, 2024; Long et al., 2021; Oliveira et al., 2017; Thompson-Booth et al., 2014a). Differently, two studies revealed that adults’ attentional bias to neutral infant faces was greater compared to the one displayed to happy and sad expressions (Jia et al., 2017, 2021).

In addition to this, a comprehensive review by Lucion et al. (2017) outlined that the attentional bias to infant faces can be influenced by parental status and sex. Therefore, even though an attentional bias to infant faces was evidenced in both non-parents (Brosch et al., 2007; Cárdenas et al., 2013) and parents (Gemignani et al., 2022, 2024; Oliveira et al., 2017; Pearson et al., 2010, 2011a, 2011b, 2013; Thompson-Booth et al., 2014a, 2014b), parents consistently showed a higher attentional bias toward infant cues (Oliveira et al., 2017; Thompson-Booth et al., 2014a, 2014b). Accordingly, hormones, social learning, together with caregiving experiences, may heighten the recruitment of attentional resources to infants in parents, ultimately fostering the parent–child bond (Parsons et al., 2017). On the other hand, evidence on sex differences generally favored females over males (Lucion et al., 2017). For instance, a study examining adults' overt attention to infant faces demonstrated a more consistent allocation to infant faces in females versus males (Cárdenas et al., 2013). A female advantage in processing infant stimuli was also showed in other behavioral (Parsons et al., 2021) and neurophysiological research (Colasante et al., 2017; Jia et al., 2022; Proverbio et al., 2006); this evidence was mainly explained in terms of the differential hormonal status and pregnancy-related changes between women and men (Hahn & Perrett, 2014). Nonetheless, other studies did not find any differences in the attentional bias to infant faces between mothers and fathers (Gemignani et al., 2022; Oliveira et al., 2017) or between nulliparous females and males (Brosch et al., 2007). Using a Go/no-Go modified attentional task (Bindemann et al., 2005), Oliveira et al. (2017) found an enhanced attention bias to infant faces in both mothers and fathers compared to non-parents. Recently, using a similar experimental paradigm, Gemignani et al. (2022) did not find any differences between mothers and fathers in the attentional bias to infant faces when the parental involvement with early childcare was considered. Altogether, evidence on sex differences in the attentional bias to infant faces has been therefore inconclusive.

For the most part, previous research on attentional bias to infant faces was confined to heteronormative samples of mothers; to date, only one research investigated this cognitive process in a sample of same-sex mother families (Gemignani et al., 2024). Moreover, no research to date has included same-sex families of men. The studies investigating more articulated parenting behaviors highlighted positive parenting qualities, such as a high level of sensitivity and responsiveness during interactions, in same-sex parent families (e.g., Carone et al., 2020; Golombok et al., 2014). Generally, these studies did not find any differences between same-sex and different-sex parents based on the type of family structure (e.g., Bos et al., 2004; Maccallum & Golombok, 2004; Rubio et al., 2017), except for those studies that found more optimal parental qualities in same-sex parents (e.g., Baiocco et al., 2015, 2018; Golombok et al., 2014). Differently, implicit processes underlying the parental response, such as the attentional bias to infant faces, have been left uninvestigated in different family forms. Given the potential implications, it would be important to enrich the understanding of those processes beyond the results found with heteronormative parent families. Moreover, considering that the attentional bias to infant faces has been associated with factors (e.g., breastfeeding, pregnancy; Dudek & Haley, 2020; Pearson et al., 2011a, 2011b) that might differ among sexual minority parent families, potential variations are worthy to be ascertained.

Overall, the current study aimed to provide new insights on the attentional bias to infant versus adult faces in a matched sample of different-sex and same-sex parents. Therefore, we explored the contribution of (1) parents’ sex and (2) family structure (i.e., different-sex and same-sex parent families) on this cognitive mechanism. Notably, although we did not expect to find any differences in the attentional bias to infant faces between same-sex and different-sex parent families, we thought that extending previous knowledge confined to heteronormative samples of parents would be needed.

First, we expected that infant faces interfered with the task performance more than adult faces, slowing RTs to peripheral stimuli in Go conditions. Given that an effect of the emotional valence of faces was found in some previous research (Pearson et al., 2010, 2011a, 2011b, 2013) but failed to be replicated in other studies (Dudek & Haley, 2020; Gemignani et al., 2022, 2024; Long et al., 2021; Oliveira et al., 2017; Thompson-Booth et al., 2014a), the potential role of the type of facial expression was further investigated in this study. Secondly, due to the inconsistent findings so far (e.g., Cárdenas et al., 2013; Gemignani et al., 2022; Oliveira et al., 2017; Proverbio et al., 2006), we could not formulate a strong a priori hypothesis on the role of sex on attentional bias to infant faces. However, since previous studies were focused on parents from different-sex families in which mothers are traditionally more involved in caretaking, we might assume that including same-sex families, in which caregiving responsibilities are more equally shared (Farr & Patterson, 2013; Patterson et al., 2004), could highlight similar attentional responses between mothers and fathers. Thirdly, considering that the type of family did not have a significant effect on more complex parental processes (e.g., Bos et al., 2004; Maccallum & Golombok, 2004; Rubio et al., 2017), we expected that it would not have any effects on the attentional bias to infant faces either. However, since no other studies examined these relationships before, we overall favored an exploratory approach.

## Method

### Participants

An initial sample of 88 parents (22 mothers and 22 fathers from same-sex parent families; 22 mothers; 22 fathers from different-sex parent families) with a child ranging between 2 and 36 months of age was considered for the study. Parents were recruited through the Italian Rainbow Family association (“Associazione Famiglie Arcobaleno”); a snowball sampling technique was used to make further contact with parents. Data on different-sex parents were taken from Gemignani et al. (2022); data on same-sex mothers were taken from Gemignani et al. (2024). Data on same-sex fathers has not been published elsewhere. A couple of two-male parents were excluded from the final sample (i.e., 86 parents; 25.6% mothers and 23.2% fathers from same-sex parent families; 25.6% mothers; 25.6% fathers from different-sex parent families) since they had a very low accuracy in performing the task. A power analysis was not performed for this study (see the difficulties in the analysis of power for linear mixed-effects models; Kumle et al., 2021), but the size of the total sample was consistent with the one adopted in previous research (e.g., Jia et al., 2021).

The four groups of parents were matched based on the child's age and socioeconomic status (SES) to rule out that group differences could be attributed to those characteristics. The SES was calculated according to Hollingshead’s (1975) criteria. The SES index showed a variation from medium (*n* = 18) to medium–high (*n* = 62) and high (*n* = 6) (Rossi, 1994). The majority of parents participated with their partner (*n* = 80), but 6 parents (4 fathers and 2 mothers from same-sex parent families) participated alone. An overview of the characteristics of study participants is reported in Table 1.

**Table 1:** Characteristics of study participants by groups

	Same-sex parents				Different-sex parents			
	Mothers (n = 22)		Fathers (n = 20)		Mothers (n = 22)		Fathers (n = 22)	
Variable	N	Mean (SD) or %	N	Mean (SD) or %	N	Mean (SD) or %	N	Mean (SD) or %
Socioeconomic status	22		20		22		22	
medium	0	0%	4	20%	7	32%	7	32%
medium–high	21	95%	13	65%	14	63%	14	63%
high	1	5%	3	15%	1	5%	1	5%
Age (in years)	22	39.6 (6.3)	20	44.4 (6.2)	22	33.6 (4.7)	22	35.1 (4.6)
Relationship status	22		20		22		22	
< 5 years	2	9%	3	15%	2	9%	2	9%
6–10 years	11	50%	2	10%	10	45%	10	45%
11–15 years	7	32%	8	40%	5	23%	5	23%
> 15 years	2	9%	7	35%	5	23%	5	23%
Number of children	22		20		22		22	
One	20	91%	15	75%	15	68%	15	68%
Two or more	2	9%	5	25%	7	32%	7	32%
Child age (in months)	22	14.0 (9.9)	20	15.3 (10.8)	22	15.2 (8.8)	22	15.3 (8.9)

### Procedure

All the procedures were conducted online. Sociodemographic information of participants was first collected through Qualtrics (Qualtrics, Provo, UT). The experimental task was run on JATOS (Lange et al., 2015). Participants performed the task during a Zoom meeting; after explaining the instructions, the experimenter shut down the microphone and camera, but they could monitor the reliability of data collected during the whole experiment.

Participants completed a modified Go/no-Go task derived from an established paradigm (Bindemann et al., 2005) to measure attentional bias to infant versus adult faces (Dudek & Haley, 2020; Gemignani et al., 2022, 2024; Pearson et al., 2010, 2011a, 2011b, 2013). Participants were asked to focus on a central fixation cross “ + ” that signaled the Go or no-Go condition by turning into a green or red font. Simultaneously, two lines, one horizontal and one vertical, appeared at the periphery of the screen. A face displaying a happy or a neutral or a sad adult or a happy or a neutral or a sad infant appeared behind the fixation Go/no-Go cross. Only for Go trials, participants indicated on which side of the screen the vertical line appeared by pressing “n” (for right) or “v” (for left) on the keyboard. The screen response was aborted if no response was registered within 2000 ms. The experimental stimuli consisted of 36 images of unfamiliar faces of infants aged 4–12 months (six males; six females) extracted from the Tromso Infant Faces Database (Maack et al., 2017) and 36 images of unfamiliar adult faces (six males; six females) taken from the Karolinska Directed Emotional Faces (Lundqvist et al., 1998). Faces were adjusted for low-level properties (luminance, saturation), cropped into an oval shape, converted into grayscale, and presented against a uniform white background. Images averaged approximately 170 × 198 pixels. Participants completed a practice block of 12 trials with no images and then a practice block of 12 trials displaying faces in the background. Experimental trials consisted of six blocks of 36 trials (24 Go and 12 no-Go). The order of trials was randomized within blocks, but Go trials occurred twice as frequently as no-Go trials. To decrease the potential risk of popout (Palermo & Rhodes, 2003), conditions were fixed in each block. Block order was randomized across subjects. The target line location was balanced within each block (50% on the right; 50% on the left). Attention was measured by calculating RTs in milliseconds (ms) to identify the location of the target vertical line from the onset of the stimulus display in Go trials. The faces that recruited greater attention resulted in slower RTs in identifying the target vertical line. Participants were asked to (1) be sitting in a quiet environment; (2) keep their left index finger on the “v” and their right index finger on the “n” of the keyboard during the task; (3) ignore the face stimuli appearing in the background; (4) be as accurate as possible in the localization judgment. Further details about the experimental procedure can be found in Gemignani et al. (2022, 2024).

### Data Analysis

At first pass, one-way ANOVAs were run to check for potential group differences (groups: mothers of same-sex parent families, fathers of same-sex parent families, mothers of different-sex parent families, fathers of different-sex parent families) in the continuous variables. Pearson's chi-square test was used to check for group differences in the relationship status. Missing data in self-reports were not replaced. In the Go/no-Go task, the overall accuracy for Go trials was 96.9%, which confirmed the ability of participants to complete the task. The percentage of false alarms (i.e., incorrect No-Go trials) was 2.5%. Response accuracy was analyzed using generalized linear mixed models (GLMMs). Then, RTs were analyzed considering only correct trials. Across all blocks, too fast responses (below 100 ms) or RTs above 1400 ms from the stimulus onset were removed (0.4%). RTs were transformed into logarithms, and the distribution was checked visually on the trial-, participant- and item- levels. As the distributions were approximately normal, we did not exclude any further items, participants, or trials. RTs were analyzed via linear mixed models performed using the lme4 library (Bates et al., 2015) in Rstudio. Effects were reported according to Type II Wald chi-square tests. Face age (adult, infant) and emotional valence (sad, neutral, happy faces) were recoded as follows: face age: “ − 1” infant, “1” adult; emotional valence: “ − 1” sad faces, “0” neutral faces, “1” happy faces. Family structure (same-sex parent family, different-sex parent family) was recoded as follows: “ − 1” same-sex parent family, “1” different-sex parent family. Sex (mothers, fathers) was recoded as follows: “ − 1” fathers, “1” mothers. Parent age was centered by subtracting the mean across all participants. To control for the potential dependency of data within the couples, we accounted for the dyad in the random structures of the models in the supplementary analyses. In addition, giventhat adult male and female faces were previously shown to affect adults’ attention differently (e.g., Hahn & Perrett, 2014), we checked the results of the models by taking the gender of adult faces into account. In particular, rather than combining adult male and female faces into a single condition, the new variable “face age (gender)” was operationalized as follows: “0” infant; “ − 1” adult male; “1” adult female. This variable was tested in the supplementary analyses.

## Results

### Preliminary Analyses

SES, child age, number of children, and relationship status did not differ among the groups. Parent age differed among the groups (*F*(3, 82) = 16.47, *p* < 0.001, *ηp2* = 0.38). Post hoc comparisons using the Tukey HSD test revealed that fathers of same-sex parent families were older as compared to all the other groups (mothers versus fathers of same-sex parent families, *diff* =  − 4.80, *p* = 0.03; mothers of different- versus fathers of same-sex parent families, *diff* =  − 10.76, *p* < 0.001; fathers of different- versus fathers of same-sex parent families, *diff* =  − 9.30, *p* < 0.001). In addition, mothers (*diff* =  − 5.95, *p* < 0.01) and fathers (*diff* =  − 4.30, *p* = 0.04) of different-sex parent families were younger compared to mothers from same-sex parent families. The means of non-log-transformed RTs (SD) as a function of face age conditions and groups are reported in Table 2.

**Table 2:** Mean RTs (SD) in ms as a function of the face age in each group

	Same-sex parents		Different-sex parents	
Face Age	Mothers (*n* = 22)	Fathers (*n* = 20)	Mothers (*n* = 22)	Fathers (*n* = 22)
Infant	687.0 (157.2) ms	715.9 (143.2) ms	687.5 (143.2) ms	637.7 (110.0) ms
Adult	663.1 (157.8) ms	696.0 (137.4) ms	672.0 (139.2) ms	630.8 (113.8) ms

*ms* milliseconds, *SD* standard deviation

### Main Analyses

First, we fitted a GLMM with face age and emotional valence predicting trial-level accuracy. The model was checked by adding parent age, parent sex, and family structure as covariates. Due to a high level of accuracy, the analyses did not yield any significant result; so, all subsequent models used RTs as dependent variable. We built Model 1 in which face age and emotional valence were used as fixed terms and their interaction was considered. Model 1 showed a main effect of face age (χ^***2***^(1, *N* = 86) = 10.44, *p* = 0.001), as infants slowed RTs to a greater extent compared to adult faces. Neither a main effect of emotional valence nor an interaction between face age and emotional valence was found.

We built Model 2 in which sex and family structure were added as fixed effects, in addition to face age. Since an effect of emotional valence did not emerge in Model 1, we collapsed across conditions. The interactions between the terms were considered. Parent age was added as a covariate. Model 2 highlighted the main effect of face age (χ^***2***^ (1, *N* = 86) = 11.14, *p* < 0.001) in the same direction as displayed by Model 1. A main effect of parent age also emerged (χ^***2***^(1, *N* = 86) = 12.17, *p* < 0.001), as older parents were overall slower to perform the task. Neither a main effect nor an interaction effect related to family structure or parents’ sex was found; in particular, no interactions between family structure and face age or parent sex and face age were detected. Further details on the main models (Model 1, Model 2) are reported in Supplementary Material 1 (SM1). Supplementary analyses also showed that the results stayed robust after considering the dependency of data within the couples in the random structures (Supplementary Material 2; SM2). Moreover, considering the gender of adult faces, we confirmed that infant faces retained more attention as compared to both female and male adult faces (Supplementary Material 3; SM3). Additional effects were not evidenced.

## Discussion

In a matched sample of different-sex and same-sex parents, our aim was to explore whether an enhanced attention to infant versus adult faces varied depending on parents’ sex and the type of family structure. In the light of previous mixed results, we did not find any differences in attentional bias to infant faces between mothers and fathers. According to our hypothesis, no effects of the type of family were found. Extending previous evidence mostly confined to heteronormative samples of parents, our work suggested that an attentional prioritization of infant faces might occur in parents independently of their sex and the type of family formation.

Preliminary findings showed that fathers of same-sex parent families were older compared to the other groups; this evidence might be related to the complicated long journey that they need to navigate to become parents (Carneiro et al., 2017). In a similar way, mothers of same-sex parent families were older compared to mothers and fathers of different-sex parent families. Adding parents’ age as a covariate to the main analyses, age consistently predicted RTs in response to both adult and infant faces. This result was consistent with the averaged RTs in different groups as displayed in Table 2; fathers of same-sex parent families had overall higher RTs compared to the other groups, due to the independent effect of age on RTs.

Consistent with the previous literature (Gemignani, 2022, 2024; Oliveira et al., 2017; Pearson et al., 2010, 2011a, 2011b, 2013; Thompson-Booth et al., 2014a, 2014b), we confirmed that infant faces retained a greater attention compared to adult faces. As shown in Table 2, the averaged RTs were consistently higher in response to infant than adult faces for all the groups, indicating the robust effect of baby schema. According to some previous evidence (Dudek & Haley, 2020; Gemignani et al., 2022, 2024; Long et al., 2021; Oliveira et al., 2017; Thompson-Booth et al., 2014a), we did not find any effects of the emotional valence of faces. Therefore, parents’ attention generally prioritized infant faces rather than adult ones, independently of the facial expression displayed. As it has been previously suggested (Dudek & Haley, 2020), while the attentional bias to infant faces might be modulated by a general preferential processing of infant faces over other social stimuli, a neural preference might be more specific to the emotional valence of faces.

Noticeably, neither parents’ sex nor the type of family structure modulated parents’ attentional bias to infant versus adult faces. First at all, it is important to note that these null findings should be read in the light of the exploratory nature of the analyses; because a power analysis was not conducted, we cannot rule out that a lack of statistically significant differences might be due to power issues. Having said that, a lack of an interaction effect between parents’ sex and face age might suggest that, despite some previous evidence indicating a maternal advantage in processing infant stimuli (e.g., Proverbio et al., 2006), the attentional prioritization of infant cues might come together in mothers and fathers alike. This evidence aligned with the idea that maternal and paternal responses might converge in some ways (Biblarz & Stacey, 2010; Fagan et al., 2014). Accordingly, whenever differences favored mothers over fathers in previous research, they possibly reflected differences in the ways males and females are socialized to parents, but not differences in parents’ sex (e.g., Ellis-Davies et al., 2022). On this note, behavioral responses have been found susceptible to social expectations related to adults’ gender and sex (Ding et al., 2020). As our sample was half composed by same-sex parents who are less susceptible to the gendered cultural norms related to parenthood (e.g., Farr & Patterson, 2013; Patterson et al., 2004), a lack of a significant effect of sex could be particularly evidenced. Differently, the conflation between sex and gendered cultural norms might be higher in the studies including heterosexual parents only (Endendijk et al., 2018a). Therefore, a proper assessment of the division of childcare tasks within the couples should be addressed in future studies aimed to corroborate these assumptions.

Regarding the non-significant effect of the family structure, strong empirical evidence has already supported that this variable is not influential for parental qualities and child developmental outcomes (Baiocco et al., 2015, 2018; Crouch et al., 2016; Fedewa et al., 2015; Golombok et al., 2018; Patterson, 2017). Nonetheless, our study was the first one focusing on an underlying cognitive process related to the human caregiving response, such as the attentional bias to infant faces. As a result, attentional bias to infant faces did not significantly vary among different types of families; in other words, the highly salient nature of infant faces seemed to be prioritized by parents’ attention independently on the type of family formation. In the light of the potential adaptive value for the quality of parent–child interactions and relationships (Dudek & Haley, 2020; Pearson et al., 2011a).

Notwithstanding the novelty of the study, our findings should be interpreted in the light of some limitations. First, our study was exploratory in nature, so the results should be interpreted as tentative. In addition, the small sample size for each group of parents limited the interpretation of our results. On this note, significant difficulties in recruiting a sample of same-sex mothers and—especially—same-sex fathers in Italy should be mentioned. In the Italian context, in fact, it is not possible to adopt children for same-sex couples. Most children in same-sex mother families are conceived through medically assisted procreation techniques, which must be accomplished abroad (e.g., in the Netherlands, Spain). When it comes to same-sex families of men, the journey to parenthood can be even more emotionally, practically, and economically challenging (Carone et al., 2021). Besides the fact that they are not able to adopt, surrogacy is prohibited in many countries across Europe, namely Austria, Finland, France, Germany, Italy, Norway, Sweden, and Switzerland, and unregulated in Belgium, the Czech Republic, Ireland, Luxembourg, and Romania (D’Amore et al., 2023). This complex situation has contributed to a very low prevalence of same-sex fathers in Italy (Baiocco et al., 2015). Nonetheless, to ensure that same-sex parents were represented in our work, the child age range adopted here was a bit large and not consistent with the age range of the infant stimuli in the Go/no-go task. A narrower child age should be hopefully adopted in future research, as this has been demonstrated to be a possible predictor of parental responsiveness to infant faces (Kuzava et al., 2020).

As another limitation, our sample included only a selected group of well-educated Caucasian parents. Therefore, a possible selection issue might have contributed to the lack of a sex effect in our study; as it was demonstrated in previous research (e.g., Craig & Mullan, 2011), more educated parents are more likely to equally share childcare duties, thus drawing an equal attention to infants. In this regard, since different countries have different views regarding the division of parental care, we cannot rule out that our results might be influenced by the cultural norms related to the Italian context. Given that these contextual differences might influence parents’ responses to infant cues, our findings should be replicated in other cultures and countries.

Furthermore, infant faces were treated here as a homogenous group, not accounting for potential differences in their levels of cuteness. To date, adult sensitivity to infant cues has been examined in two ways: by comparing responses to infant versus adult face or by manipulating the cuteness of infant faces. In accordance with some recent evidence (Endendijk et al., 2018b), the first approach was adopted in the present study. However, given the importance of infant cuteness in modulating adults’ processing of infant cues (e.g., Glocker et al., 2009; Hahn et al., 2016; Kringelbach et al., 2016; Zhang et al., 2012), future research should use an experimental design in which infant faces are manipulated for cuteness (Hahn et al., 2016). Even though the supplementary analyses suggested that no effects of adult face attractiveness—based on the gender—emerged in our study (e.g., see Hahn & Perrett, 2014), this evidence should be corroborated in future studies by manipulating facial attractiveness. In addition, as behavioral measures can be also influenced by adults’ interest in providing care for infants (Hahn et al., 2015), an assessment of motivational tendencies in caregiving should be also included in future.

Moreover, behavioral measures such as RTs reflect a considerable number of cognitive processes that take place and get consolidated by the time the individual makes a response; however, they cannot provide any information regarding the separated stages of attention deployment toward a specific stimulus. Differently, EEG paradigms can better reveal the time course of the adults’ attentional bias toward infant faces (Torrence & Troup, 2018). As implicit neural measures such as EEG might be also less sensitive to social expectations and biases (e.g., Ding et al., 2020), they should be importantly adopted in future. Unfortunately, as this experiment was conducted by the time of COVID-19 pandemics in Italy, we had a limited access to laboratory-based measures. In addition, all the procedures were conducted online to overcome geographical barriers in recruiting the sample; this aspect was particularly relevant for the recruitment of same-sex parents, who were not required to travel to our laboratory from different regions of Italy to join the research.

Remarkably, viariations in the attentional bias to infant faces have been associated with the quality of caregiving behaviors in previous research (Dudek & Haley, 2020; Pearson et al., 2010, 2013). Given that we did not assess parental behaviors in the present work, future research should elucidate the ecological validity of our findings by drawing a line between the measure of attentional bias to infant faces and the quality of parental behaviors in different families. Even though we acknowledge the value of our experimental approach in teasing apart how infant faces are processed by parents, the practical applications of it should be importantly understood and optimized.

### Conclusion

In conclusion, the present work provided a more comprehensive understanding of the adults’ cognitive responses to infant faces going beyond a heteronormative perspective on parenting. In general, a broader perspective on parenting that takes into account family diversities might shed further light on the underlying mechanisms that promote human caregiving responses. Not least of all, much research including different types of families would socialize the idea that there are different ways of perceiving and understanding parenthood in this area of research. Inclusive data involving LGBTQ+ parents would potentially help to improve the family outcomes and reduce disparities.

### Supplementary Information

Below is the link to the electronic supplementary material.

Supplementary file1 (DOCX 20 KB)

Supplementary file2 (DOCX 19 KB)

Supplementary file3 (DOCX 18 KB)

## Data Availability

The data and codes are not publicly available, but only upon request to the corresponding author.
