# Supplementary material for: Neither Parents’ Sex Nor the Type of Family Modulates Attentional Bias Toward Infant Faces: A Preliminary Study in Different-Sex and Same-Sex Parents
Source: Arch Sex Behav. 2024 May 29;53(6):2053–61. doi: 10.1007/s10508-024-02875-9 (PMC11176217; doi:10.1007/s10508-024-02875-9)
Supplement: Supplementary file 2 — Supplementary file2 (DOCX 19 KB) [file 10508_2024_2875_MOESM2_ESM.docx]

**Model 1**: *logRT ~ Face age * Emotional valence + (1 + Face age * Emotional valence | subject) + (1 | stimuli) + (1 | dyad)*

| **Fixed Effects** | | | |
| --- | --- | --- | --- |
| **Effects** | χ***^2^*** | **Df** | ***p*-value** |
| Face age | 10.44 | 1 | 0.001** |
| Emotional valence | 0.24 | 2 | 0.9 |
| Face age*Emotional valence | 1.49 | 2 | 0.48 |

*Note. *** = p < .001; ** = p < .01; * = p < .05*

| **Random Effects** | | | |
| --- | --- | --- | --- |
| **Groups** | **Name** | **Variance** | **SD** |
| Subject | (Intercept) | 0.02 | 0.15 |
|  | Face age (adult) | 0.009 | 0.1 |
|  | Emotional Valence (neutral) | 0.006 | 0.08 |
|  | Emotional Valence (happy) | 0.007 | 0.09 |
|  | Face age (adult)*Emotional valence (neutral) | 0.01 | 0.12 |
|  | Face age (adult)*Emotional valence (happy) | 0.02 | 0.13 |
| Stimuli | (Intercept) | 0.0001 | 0.01 |
| Dyad | (Intercept) | 0.003 | 0.05 |
| Residual |  | 0.02 | 0.13 |

**Model 2**: *logRT ~ Face age * Sex * Family Structure + Parent Age + (1+ Face age | subject) + (1 + Sex * Family Structure + Parent Age | stimuli) + (1 | dyad)*

| **Fixed Effects** | | | |
| --- | --- | --- | --- |
| **Effects** | χ***^2^*** | **Df** | ***p*-value** |
| Face age | 11.10 | 1 | <0.0001*** |
| Sex | 1.89 | 1 | 0.17 |
| Family Structure | 0.76 | 1 | 0.38 |
| Parent Age | 11.26 | 1 | <0.0001*** |
| Face age*Sex | 0.83 | 1 | 0.36 |
| Face age*Family Structure | 1.09 | 1 | 0.30 |
| Sex*Family Structure | 1.85 | 1 | 0.17 |
| Face age*Sex*Family Structure | 0.002 | 1 | 0.97 |

*Note. *** = p < .001; ** = p < .01; * = p < .05*

| **Random Effects** | | | |
| --- | --- | --- | --- |
| **Groups** | **Name** | **Variance** | **SD** |
| Subject | (Intercept) | 0.02 | 0.13 |
|  | Face (adult) | 0.004 | 0.06 |
| Stimuli | (Intercept) | 0.0003 | 0.02 |
|  | Sex (female) | 0.0002 | 0.01 |
|  | Family Structure (different-sex parent family) | 0.0002 | 0.02 |
|  | Parent Age | 0.000001 | 0.001 |
|  | Sex (female)  *Family Structure (different-sex parent family) | 0.0002 | 0.02 |
| Dyad | (Intercept) | 0.002 | 0.05 |
| Residual |  | 0.02 | 0.14 |
