# Supplementary material for: Neither Parents’ Sex Nor the Type of Family Modulates Attentional Bias Toward Infant Faces: A Preliminary Study in Different-Sex and Same-Sex Parents
Source: Arch Sex Behav. 2024 May 29;53(6):2053–61. doi: 10.1007/s10508-024-02875-9 (PMC11176217; doi:10.1007/s10508-024-02875-9)
Supplement: Supplementary file 3 — Supplementary file3 (DOCX 18 KB) [file 10508_2024_2875_MOESM3_ESM.docx]

**Model 1**: *logRT ~ Face age (gender) * Emotional valence + (1 + Face age (gender) * Emotional valence | subject) + (1 | stimuli)*

| **Fixed Effects** | | | |
| --- | --- | --- | --- |
| **Effects** | χ***^2^*** | **Df** | ***p*-value** |
| Face age (gender) | 15.35 | 2 | <0.001*** |
| Emotional valence | 0.25 | 2 | 0.9 |
| Face age (gender) *Emotional valence | 2.01 | 4 | 0.73 |

*Note. *** = p < .001; ** = p < .01; * = p < .05*

| **Post-hoc comparisons** | | | | |
| --- | --- | --- | --- | --- |
| **Contrasts** | **Estimate** | **SE** | **z ratio** | **p value** |
| Adult male - Infant | -0.02 | 0.008 | -2.43 | 0.04* |
| Adult male - Adult female | 0.01 | 0.005 | 2.18 | 0.07 |
| Infant - Adult female | 0.03 | 0.008 | 3.78 | <0.0001*** |

*Note. *** = p < .001; ** = p < .01; * = p < .05*

**Model 2**: *logRT ~ Face age (gender) * Sex * Family Structure + Parent Age + (1+ Face age (gender) | subject) + (1 + Sex * Family Structure + Parent Age | stimuli)*

| **Fixed Effects** | | | |
| --- | --- | --- | --- |
| **Effects** | χ***^2^*** | **Df** | ***p*-value** |
| Face age (gender) | 15.76 | 2 | <0.0001*** |
| Sex | 1.10 | 1 | 0.30 |
| Family Structure | 0.91 | 1 | 0.34 |
| Parent Age | 12.04 | 1 | <0.0001*** |
| Face age (gender)*Sex | 2.04 | 2 | 0.36 |
| Face age (gender)*Family Structure | 1.38 | 2 | 0.50 |
| Sex*Family Structure | 1.76 | 1 | 0.19 |
| Face age*Sex*Family Structure | 1.02 | 2 | 0.60 |

*Note. *** = p < .001; ** = p < .01; * = p < .05*

| **Post-hoc comparisons** | | | | |
| --- | --- | --- | --- | --- |
| **Contrasts** | **Estimate** | **SE** | **z ratio** | **p value** |
| Adult male - Infant | -0.02 | 0.008 | -2.44 | 0.04* |
| Adult male - Adult female | 0.01 | 0.005 | 2.22 | 0.07 |
| Infant - Adult female | 0.03 | 0.008 | 3.95 | <0.0001*** |
